# Supplementary material for: Re-assessment of net energy production and greenhouse gas emissions avoidance after 40 years of photovoltaics development
Source: Nat Commun. 2016 Dec 6;7:13728. doi: 10.1038/ncomms13728 (PMC5150644; doi:10.1038/ncomms13728)
Supplement: Supplementary Information — Supplementary Tables, Supplementary Methods, Supplementary References [file ncomms13728-s1.pdf]

SUPPLEMENTARY TABLES

Supplementary Table 1. Data for cumulative energy demand and energy payback time of multicrystalline silicon solar cells, modules and systems. Energy payback time was calculated from CED with a harmonised procedure, assuming an insolation of 1700 $kWhm^2yr^{-1}$ and a performance ratio of 0.75. Studies that did not present results for full systems, or that present results for worst case or best case scenarios, as well as prospective studies were excluded from our analysis.

**Reference**	**Data Year**	**Cumulative Energy Demand ($MJ_P\ W_p^{-1}$)**			**EPBT (years)**			**Excluded**	**Prospective**
		Cells	Modules	Systems	Cells	Modules	Systems		
[1]	1976	246			16.7			•	
[1]	1976	510			34.6			•	
[2]	1977	93.6	96.1		6.34	6.51		•	
[3]	1977		270	637		18.3	43.1	•	
[4]	1985	166			11.3			•	
[5]	1989		59.4	72.0		4.02	4.88		
[6]	1993	60.6	63.2	68.4	4.10	4.28	4.63		
[7]	1996	24.3	33.0		1.65	2.24		•	
[5]	1997		27.0	32.4		1.83	2.20	•	•
[8]	1997	23.9	30.5		1.62	2.06		•	
[8]	1997	22.5	28.9		1.52	1.96		•	
[8]	1997	10.7	14.4		0.73	0.98		•	•
[9]	1997	70.3	93.1		4.76	6.31		•	
[9]	1997	24.1	36.2		1.63	2.45		•	
[10]	1998		36.8			2.49		•	
[9]	1999	25.7	36.2		1.74	2.45		•	
[11]	1999	29.6	36.2	57.9	2.01	2.45	3.92		
[11]	1999	26.9	35.4	41.8	1.82	2.40	2.83		
[12]	2003		34.0			2.30		•	
[13]	2005	22.0	29.8	32.5	1.49	2.02	2.20		
[14]	2006			32.5			2.20		
[15]	2005		25.6	35.3		1.73	2.39		
[16]	1999	43.1	48.1	53.2	2.92	3.26	3.60		
[17]	2006		16.9	32.5		1.14	2.21		
[18]	1999			49.2			3.34		
[15]	2007		69.4	78.6		4.70	5.33	•	
[15]	2007		25.3	34.4		1.71	2.33		
[19]	2008			33.1			2.24		
[20]	2008		16.0	31.3		1.08	2.12		
[21]	2009			23.5			1.59		
[22]	2009		19.7	23.9		1.33	1.62		
[11]	2010		17.3	21.6		1.17	1.46	•	•
[23]	2011	6.25	14.8	15.8	0.42	1.00	1.07		
[23]	2011	6.17	17.9	18.9	0.42	1.21	1.28		
[24]	2013	3.71	7.82	13.7	0.25	0.530	0.930		
[11]	2020		12.9	16.7		0.877	1.13	•	•

Supplementary Table 2. Data for cumulative energy demand and energy payback time of mono-crystalline silicon solar cells, modules and systems. Energy payback time was calculated from CED with a harmonised procedure, assuming an insolation of 1700 $kWhm^2yr^{-1}$ and a performance ratio of 0.75. Studies that did not present results for full systems, or that present results for worst case or best case scenarios, as well as prospective studies were excluded from our analysis.

**Reference**	**Data Year**	**Cumulative Energy Demand ($MJ_P\ W_p^{-1}$)**			**EPBT (years)**			**Excluded**	**Prospective**
		Cells	Modules	Systems	Cells	Modules	Systems		
[5]	1989		63.0	73.8		4.32	5.06		
[6]	1993	57.4	59.6	63.3	3.94	4.09	4.34		
[25]	1995		63.1			4.33		•	
[25]	1995		11.3			0.779		•	
[5]	1997		39.6	43.9		2.72	3.01	•	•
[26]	1997			63.2			4.34		
[8]	1997	123	127		8.45	8.73		•	
[8]	1997	91.5	95.7		6.28	6.57		•	
[8]	1997	29.9	34.1		2.05	2.34		•	•
[9]	1997	44.5	49.6	53.5	3.05	3.40		•	
[9]	1997	107	116	123	7.32	7.93		•	
[10]	1998		63.7			4.37		•	
[27]	1999	52.2	64.0		3.58	4.39		•	
[11]	1999		44.3	58.1		3.04	3.99		
[11]	1999		44.3	50.3		3.04	3.45		
[28]	2003		31.9	45.2		2.19	3.10		
[28]	2003		31.9	56.0		2.19	3.84		
[14]	2005			39.8			2.70		
[29]	2005	37.8	41.3		2.59	2.83		•	
[18]	1999			56.4			3.87		
[30]	2006		37.1	39.8		2.55	2.73		
[19]	2008			41.9			2.88		
[21]	2009			23.3			1.60		
[22]	2009		27.9	32.0		1.91	2.20		
[11]	2010		40.7	44.9		2.79	3.08	•	•
[11]	2010		38.0	42.2		2.61	2.90	•	•
[23]	2011	13.4	25.3	26.2	0.92	1.74	1.80		
[23]	2011	13.2	30.8	31.7	0.90	2.11	2.18		
[31]	2012	16.3	21.2	29.2	1.12	1.45	2.00		
[24]	2013	5.15	10.0	16.1	0.35	0.684	1.10		
[11]	2020		33.5	37.0		2.30	2.54	•	•
[11]	2020		33.5	37.0		2.30	2.54	•	•
[31]	2020	6.87	10.3	17.1	0.47	0.708	1.17	•	•
[32]	2020		9.23			0.633		•	•
[32]	2020		7.14			0.490		•	•
[32]	2020		7.00			0.480		•	•

Supplementary Table 3. Data for life cycle greenhouse gas emissions of multi-crystalline silicon PV systems. Harmonised system GHG was calculated from the reported values assuming an insolation of 1700 $kWhm^2yr^{-1}$, a performance ratio of 0.75, a lifetime of 30 years and a degradation rate of 0.7% per year. Studies that did not present results for full systems, or that present results for worst case or best case scenarios, as well as prospective studies were excluded from our analysis.

**Reference**	**Data Year**	**Reported GHG (gCO_2-eq/kWh)**				**Harmonised system GHG**		**Excluded**	**Prospective**
		Cells	Modules	BOS	System	(gCO_2-eq/kWh)	($kgCO_2$-eq/Wp)		
[5]	1989				245	143	4.90		
[5]	1989				123	143	4.90		
[33]	1991				188	94.9	3.25		
[25]	1995				305	178	6.10	•	
[25]	1995				245	143	4.90	•	
[25]	1995				179	178	6.09	•	
[25]	1995				144	143	4.90	•	
[34]	1993	42.9	55.4	58.3	114	118	4.04		
[34]	1993	58.8	76.0	67.6	144	109	3.72		
[34]	1993	57.5	73.1	59.0	132	137	4.69		
[9]	1997				60.0	67.0	2.30		
[9]	1997				150	168	5.74		
[35]	1992				189	142	4.88		
[8]	1998				73.3	49.5	1.70	•	
[8]	1998				66.0	44.6	1.53	•	
[36]	1998				87.0	87.0	2.98		
[11]	1999				60.0	67.0	2.30		
[16]	1996					138.2	4.73	•	
[14]	2005				36.4	40.7	1.39		
[30]	2006				35.0	39.1	1.34		
[15]	2005		52.0	20.0	72.0	53.6	1.84		
[37]	2005				53.0	61.0	2.09		
[38]	2007				29.5	29.5	1.01		
[11]	2010				30.0	33.5	1.15	•	•
[23]	2011	14.0	21.7	5.5	27.2	27.7	0.95		
[23]	2011	35.5	43.6	5.5	49.1	50.0	1.71	•	
[24]	2013				28.0	19.0	0.65		
[24]	2013				26.0	18.5	0.63		
[24]	2013				21.0	20.4	0.70		
[24]	2013				18.0	18.7	0.64		
[11]	2020				20.0	22.3	0.77	•	•

Supplementary Table 4. Data for life cycle greenhouse gas emission of mono-crystalline silicon PV systems. Harmonised system GHG was calculated from the reported values assuming an insolation of 1700 $kWhm^2yr^{-1}$, a performance ratio of 0.75, a lifetime of 30 years and a degradation rate of 0.7% per year. Studies that did not present results for full systems, or that present results for worst case or best case scenarios, as well as prospective studies were excluded from our analysis.

**Reference**	**Data Year**	**Reported GHG (gCO_2-eq/kWh)**				**Harmonised system GHG**		**Excluded**	**Prospective**
		Cells	Modules	BOS	System	gCO_2-eq/kWh	$kgCO_2$-eq/Wp		
[3]	1983		123		289	409	14.0		
[3]	1986				43	60	2.1	•	
[3]	1986				39	56	1.9	•	
[39]	1992				334	225	7.7		
[39]	1992				238	161	5.5		
[39]	1992				77	52	1.8	•	•
[35]	1992				189	142	4.9		
[8]	1998				304	206	7.0		
[8]	1998				224	151	5.2		
[14]	2005				45	50	1.7		
[30]	2006				45	50	1.7		
[20]	2008				44	61	2.1		
[20]	2008				34	48	1.6		
[38]	2008				30	30	1.0		
[23]	2011	26	33	5	38	41	1.4		
[23]	2011	69	76	5	81	88	3.0	•	
[31]	2012				38	42	1.5		
[24]	2013				36	24	0.8		
[24]	2013				33	23	0.8		
[24]	2013				26	25	0.9		
[24]	2013				24	24	0.8		
[31]	2020				25	28	1.0	•	•

SUPPLEMENTARY METHODS

Data filtering

Data filtering was performed on the data gathered from the LCA studies cited in Supplementary Tables 1-5, to exclude studies that in our opinion do not reflect the state of PV production corresponding with the reported data ages. In order to maximise the available datapoints, we however only exclude those results that:

Are specifically mentioned to be valid for worst case or best case scenarios

Are specifically mentioned to refer to prospective studies

Are not for complete PV systems, but only for parts (e.g. only cells or modules).

SUPPLEMENTARY REFERENCES

---

[1] Hunt, L. P. Total energy use in the production of silicon solar cells from raw materials to finished product. In *12th Photovoltaic Specialists Conference*, 347–352 (IEEE, 1976).

[2] Lindmayer, J. Solar breeder: Energy payback time for silicon photovoltaic systems. Report No. SX/111/10, (Solarex Corp, 1977).

[3] Kreith, F., Norton, P. & Brown, D. A comparison of CO 2 emissions from fossil and solar power plants in the United States. *Energy* **15**, 1181–1198 (1990).

[4] Aulich, H. A., Schulze, F. W. & Strake, B. Energy pay-back time for crystaline silicon photovoltaic modules using new technologies. In *IEEE Photovoltaic Specialists Conference. 18*, 1213–1218 (IEEE, 1985).

[5] Schaefer, H. & Hagedorn, G. Hidden energy and correlated environmental characteristics of PV power generation. *Renewable Energy* **2**, 159–166 (1992).

[6] Wetzel, T., Baake, E. & Mühlbauer, A. Energy requirements and ways of saving energy in the production process of photovoltaic modules. *International Journal of Solar Energy* **20**, 185–196 (2000).

[7] Alsema, E. A. *Environmental aspects of solar cell modules: summary report.* Report No. 96074 (Dept. of Science, Technology and Society, Utrecht University, 1996).

[8] Kato, K., Murata, A. & Sakuta, K. Energy pay-back time and life-cycle CO2 emission of residential PV power system with silicon PV module. *Progress in Photovoltaics: Research and Applications* **6**, 105–115 (1998).

[9] Alsema, E. A., Frankl, P. & Kato, K. Energy pay-back time of photovoltaic energy systems: Present status and prospects. In *2nd World Conference on Photovoltaic Solar Energy Conversion, Vienna*, 6–10 (1998).

[10] Krauter, S. & Rüther, R. Considerations for the calculation of greenhouse gas reduction by photovoltaic solar energy. *Renewable Energy* **29**, 345–355 (2004).

[11] Alsema, E. A. Energy pay-back time and CO2 emissions of PV systems. *Progress in photovoltaics: research and applications* **8**, 17–25 (2000).

[12] Meijer, A., Huijbregts, M. A. J., Schermer, J. J. & Reijnders, L. Life-cycle assessment of photovoltaic modules: Comparison of mc-Si, InGaP and InGaP/mc-Si solar modules. *Progress in Photovoltaics: Research and Applications* **11**, 275–287 (2003).

[13] Alsema, E. & de Wild, M. J. Environmental impact of crystalline silicon photovoltaic module production. In *MRS Proceedings*, vol. 895, 0895–G03 (Cambridge Univ Press, 2005).

[14] Fthenakis, V. & Alsema, E. Photovoltaics energy payback times, greenhouse gas emissions and external costs: 2004–early 2005 status. *Progress in Photovoltaics: Research and Applications* **14**, 275–280 (2006).

[15] Raugei, M., Bargigli, S. & Ulgiati, S. Life cycle assessment and energy pay-back time of advanced photovoltaic modules: CdTe and CIS compared to poly-Si. *Energy* **32**, 1310–1318 (2007).

[16] Battisti, R. & Corrado, A. Evaluation of technical improvements of photovoltaic systems through life cycle assessment methodology. *Energy* **30**, 952–967 (2005).

[17] Koroneos, C., Stylos, N. & Moussiopoulos, N. LCA of Multicrystalline Silicon Photovoltaic Systems - Part 1: Present Situation and Future Perspectives (8 pp). *The International Journal of Life Cycle Assessment* **11**, 129–136 (2006).

[18] Bankier, C. & Gale, S. *Energy Payback of Roof Mounted Photovoltaic Cells* http://www.resilience.org/stories/2006-06-16/energy-payback-roof-mounted-photovoltaic-cells (2006).

[19] Ito, M., Komoto, K. & Kurokawa, K. Life-cycle analyses of very-large scale PV systems using six types of PV modules. *Current Applied Physics* **10**, S271–S273 (2010).

[20] Ito, M., Kato, K., Komoto, K., Kichimi, T. & Kurokawa, K. A comparative study on cost and life-cycle analysis for 100 MW very large-scale PV (VLS-PV) systems in deserts using m-Si, a-Si, CdTe, and CIS modules. *Progress in Photovoltaics: Research and Applications* **16**, 17–30 (2008).

[21] Raugei, M., Fullana-i Palmer, P. & Fthenakis, V. M. The energy return on energy investment (EROI) of photovoltaics: Methodology and comparisons with fossil fuel life cycles. *Energy Policy* **45**, 576–582 (2012).

[22] Ito, M., Kudo, M., Nagura, M. & Kurokawa, K. A comparative study on life cycle analysis of 20 different PV modules installed at the Hokuto mega-solar plant. *Progress in Photovoltaics: Research and Applications* **19**, 878–886 (2011).

[23] de Wild-Scholten, M. J. Energy payback time and carbon footprint of commercial photovoltaic systems. *Solar Energy Materials and Solar Cells* **119**, 296–305 (2013).

[24] Wetzel, T. & Borchers, S. Update of energy payback time and greenhouse gas emission data for crystalline silicon photovoltaic modules: Broader perspectives. *Progress in Photovoltaics: Research and Applications* (In press) (2014).

[25] Bates, J., Watkiss, P. & Thorpe, T. *Life cycle emissions from renewable energy technologies*. Report No. XA9846090 (ETSU, 1997).

[26] Gürzenich, D., Mathur, J., Bansal, N. K. & Wagner, H.-J. Cumulative energy demand for selected renewable energy technologies. *The International Journal of Life Cycle Assessment* **4**, 143–149 (1999).

[27] Knapp, K. & Jester, T. An empirical perspective on the energy payback time for photovoltaic modules. In *Proceedings of the Solar 2000 Conference*, 641–648 (American Solar Energy Society; American Institute of Architects, 2000).

[28] Nawaz, I. & Tiwari, G. Embodied energy analysis of photovoltaic (PV) system based on macro- and micro-level. *Energy Policy* **34**, 3144–3152 (2006).

[29] Boyd, S. & Dornfeld, D. A. *Technology choices for the PV Industry: A comparative life cycle assessment.* https://escholarship.org/uc/item/9fh0w31m (Laboratory for Manufacturing and Sustainability, UC Berkely, 2005).

[30] Alsema, E. A., de Wild-Scholten, M. J. & Fthenakis, V. M. Environmental impacts of PV electricity generation-a critical comparison of energy supply options. In *21st European Photovoltaic Solar Energy Conference, Dresden, Germany*, vol. 3201 (WIP-Munich, 2006).

[31] Louwen, A., van Sark, W., Schropp, R., Turkenburg, W. & Faaij, A. Life-cycle greenhouse gas emissions and energy payback time of current and prospective silicon heterojunction solar cell designs. *Progress in Photovoltaics: Research and Applications* **23**, 1406–1428 (2015).

[32] Mann, S. A., de Wild-Scholten, M. J., Fthenakis, V. M., van Sark, W. G. & Sinke, W. C. The energy payback time of advanced crystalline silicon PV modules in 2020: A prospective study. *Progress in Photovoltaics: Research and Applications* **22**, 1180–1194 (2014).

[33] Yamada, K., Komiyama, H., Kato, K. & Inaba, A. Evaluation of photovoltaic energy systems in terms of economics, energy and CO 2 emissions. *Energy conversion and management* **36**, 819–822 (1995).

[34] Komiyama, H., Yamada, K., Inaba, A. & Kato, K. Life cycle analysis of solar cell systems as a means to reduce atomospheric carbon dioxide emissions. *Energy conversion and management* **37**, 1247–1252 (1996).

[35] Dones, R. & Frischknecht, R. Life-cycle assessment of photovoltaic systems: Results of Swiss studies on energy chains. *Progress in Photovoltaics Research and Applications* **6**, 117–125 (1998).

[36] Alsema, E. A. & Nieuwlaar, E. Energy viability of photovoltaic systems. *Energy policy* **28**, 999–1010 (2000).

[37] Hondo, H. Life cycle GHG emission analysis of power generation systems: Japanese case. *Energy* **30**, 2042–2056 (2005).

[38] Fthenakis, V. M., Kim, H. C., Held, M., Raugei, M. & Krones, J. Update of PV energy payback times and life-cycle greenhouse gas emissions. In *24th European Photovoltaic Solar Energy Conference and Exhibition*, 21–25 (WIP-Munich, 2009).

[39] Kato, K., Murata, A. & Sakuta, K. An evaluation on the life cycle of photovoltaic energy system considering production energy of off-grade silicon. *Solar Energy Materials and Solar Cells* **47**, 95–100 (1997).
